# Successful treatment with traditional Japanese medicine (*kampo* medicine) Yokukansan as a migraine prophylactic drug: A case report

**DOI:** 10.1097/MD.0000000000039072

**Published:** 2024-07-26

**Authors:** Hisanao Akiyama, Yasuhiro Hasegawa, Yoshihisa Yamano

**Affiliations:** a Department of Neurology, St. Marianna University School of Medicine, Kanagawa, Japan; b Department of Neurology, Shin-Yurigaoka General Hospital, Kanagawa, Japan.

**Keywords:** *kampo* medicine, migraine, traditional Japanese medicine, Yokukansan

## Abstract

**Rationale:**

The use of anti-CGRP antibody drugs as migraine preventive drugs is increasing worldwide, but there are still a certain number of cases where antibody drugs are ineffective or cannot be used due to high prices. Conventional prophylactic drugs or traditional Japanese medicine (*kampo* medicine) are still often used in such cases. However, to date, only limited evidence supports the efficacy of *kampo* medicine for headaches because these treatments have been used primarily empirically and traditionally. However, in recent years studies have begun to be published that describe the efficacy of *kampo* medicine for various types of headache. Here, we report the case of a patient who achieved a marked reduction in migraine frequency and severity by prophylactic therapy with the *kampo* drug yokukansan (TSUMURA Yokukansan Extract Granules).

**Patient concerns and diagnoses:**

The patient was a 50-year-old woman. She began to experience headaches around high school age and was diagnosed with migraine without aura at 42 years of age.

**Interventions and outcomes:**

She started prophylactic therapy with amitriptyline and topiramate and this treatment reduced the frequency of migraines for several years. However, the frequency began to increase again around 47 years, which is when she presented at our hospital. We achieved a temporary reduction in migraine frequency by adjusting the dose of drugs in her prophylactic therapy regimen, but the frequency increased again around age 49. We then tried monotherapy with the *kampo* medicine yokukansan, and this markedly reduced migraine frequency and severity over the following year. This therapy has remained effective to date.

**Lessons:**

We speculate that, in this case, migraine without aura was improved by prophylactic therapy with yokukansan due to its action on the glutamatergic system or serotonin system through suppression of orexin-A secretion or its anti-inflammatory effects as reported in previous animal studies. Yokukansan could be a usable *kampo* medicine for migraine prophylaxis in countries all over the world and should be investigated in a large clinical trial as soon as possible.

## 1. Introduction

Beta-blockers, calcium channel blockers, anticonvulsants, and antidepressants were used mainly as migraine-preventive drugs in the past. However, these drugs have not been widely used because they are ineffective or prone to cause side effects, and even if they are effective, the effects take time.^[1]^ In recent years, anti-CGRP antibody drugs that have solved these problems have been launched in Japan from 2021, creating a paradigm shift in migraine preventive therapy. When migraine patients are resistant to those antibody drugs or cannot use them due to high prices, however, it seems that traditional Japanese medicine (kampo medicine) may still be beneficial in addition to conventional prophylactic drugs.

The Japanese Headache Society Clinical Practice Guideline for Headache Disorders 2021 states that *kampo* medicine is effective and safe for the treatment of various types of headache, and *kampo* drugs have been used for the prevention and acute relief of headache for many years.^[2]^ It also gives a grade B recommendation to 5 *kampo* drugs: goshuyuto (evodia decoction), keishininjinto (cinnamon twig and ginseng decoction), chotosan (uncaria decoction), kakkonto (kudzu decoction) and goreisan (five-ingredient powder with poria).^[2–17]^ In recent years, a growing number of studies investigating the efficacy of *kampo* drugs for various types of headaches have been published, but our previous chronic migraine and another chronic migraine with medication overuse headache case reports are the only one specifically discussing yokukansan (TSUMURA Yokukansan Extract Granules produced by Tsumura Co. Ltd. Tokyo, Japan) to be published in an international journal.^[18,19]^

Here, we report a case in which yokukansan treatment was effective in preventing migraine without aura, specifically menstrual migraines. We also present a review of the literature.

## 2. Case presentation

**Figure 1.**
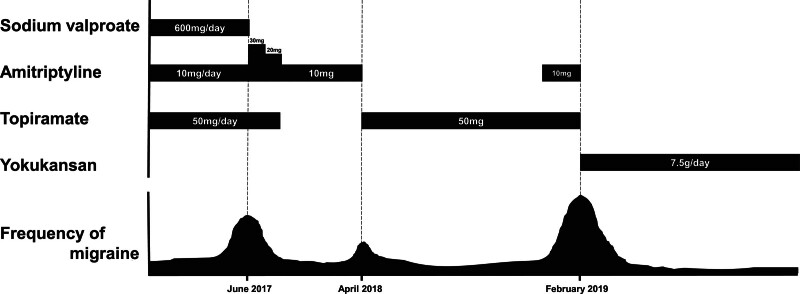
Clinical course. Prophylactic therapy with the *kampo* drug yokukansan markedly reduced migraine frequency and severity, and its effects were persistent.

The patient was a 50-year-old nurse. She came to our hospital at age 47 with a main complaint of increased frequency of headaches. Her medical history was unremarkable. She began experiencing headaches as a high school student. At the time, they manifested as throbbing headaches without aura accompanied by vomiting that was so severe that she could not move whenever they occurred. Therefore, every time she had an attack, she would go to a dark unlit place, lie down to rest without taking any analgesics, and wait for the headache to resolve. When she became a nurse at 20, the frequency of headaches increased. To prevent headaches from interfering with her work, she began frequently using over-the-counter analgesics to the point of abuse. At 42 years of age, she visited a hospital and was diagnosed with migraine without aura based on normal findings on non-contrast head MRI and the International Classification of Headache Disorders (ICHD) criteria. She started with sodium valproate 600 mg/day to prevent migraines, but it was not very effective. Amitriptyline 10 mg/day and topiramate 50 mg/day were added, and the frequency of migraines decreased. The frequency of headaches remained low for a while as she continued this preventive regimen and used a triptan as needed. However, around June 2017 (at age 47), she noticed an increase in migraine frequency during menstruation or after changes in weather or atmospheric pressure and presented to our hospital. We diagnosed migraine without aura based on normal neurological findings and diagnostic criteria in the third edition of ICHD (ICHD-3) at the visit. We discontinued sodium valproate 600 mg/day she had been taking, continued topiramate 50 mg/day, and increased the dose of amitriptyline from 10 mg to 30 mg. With these changes, the frequency of migraines decreased after 3 months. Therefore, we reduced the dose of amitriptyline to 10 mg and stopped topiramate at 50 mg/day. However, the frequency of migraines increased again around April 2018. We discontinued amitriptyline and resumed topiramate 50 mg/day, and the frequency of headaches decreased to the same level as before. Later, around January 2019, the frequency of migraines increased once again and we added amitriptyline 10 mg, but it was not effective. Therefore, in February we discontinued both topiramate 50 mg/day and amitriptyline, and started monotherapy with the *kampo* drug yokukansan at 7.5 g/day, because that anti-CGRP antibody drugs had not been launched yet in Japan (Fig. 1). When the patient started yokukansan therapy, her score on the headache impact test (HIT)-6 was 66 points. About 2 years have passed since she started yokukansan therapy. Her menstrual migraines have disappeared completely, and her migraines associated with changes in weather and atmospheric pressure have been successfully managed by taking a triptan as needed. She has not experienced any other migraines besides those, and her HIT-6 score decreased to 42 points and has remained at that level.

## 3. Discussion

**Figure 2.**
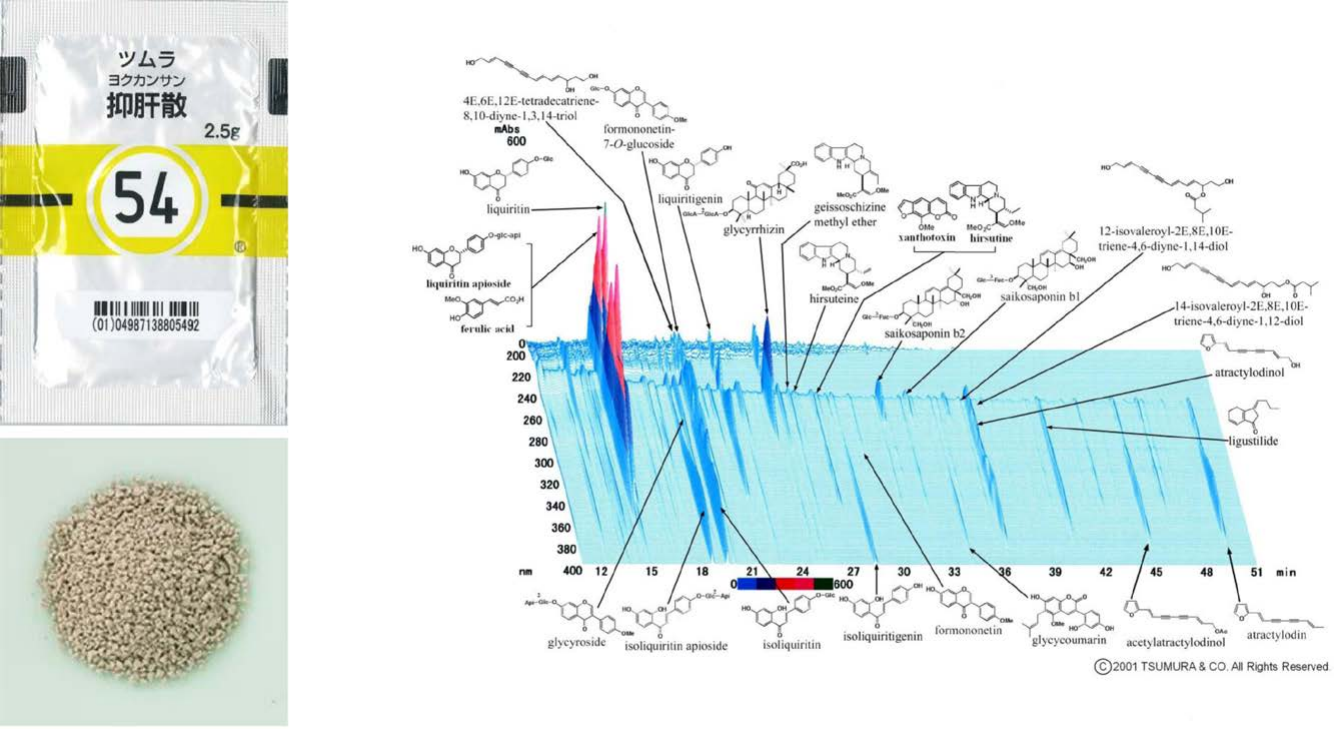
Appearance and 3D HPLC pattern of yokukansan (TSUMURA Yokukansan Extract Granules) Yokukansan appears as light grayish-brown granules. Each 7.5 g of yokukansan contains 4.25 g of excipients plus 3.25 g of dried extracts of 7 crude drugs: 2.0 g bupleurum root, 3.0 g uncaria hook, 4.0 g atractylodes lancea rhizome, 4.0 g poria sclerotium, 3.0 g Japanese angelica root, 3.0 g cnidium rhizome, and 1.5 g glycyrrhiza. 3D HPLC = three-dimensional high-performance liquid chromatographic fingerprint.

Traditional Japanese medicine (*kampo* medicine) is prepared from combinations of multiple crude drugs such as plants and minerals found in nature. The useful combinations of these crude drugs and the effects they can achieve without causing adverse events have been determined through thousands of years of treatment experience. Yokukansan (TSUMURA Yokukansan Extract Granules), one of those *kampo* drugs, consists of light grayish-brown granules. Each 7.5 g of yokukansan contains 4.25 g of excipients plus 3.25 g of dried extracts of 7 crude drugs: 2.0 g of bupleurum root, 3.0 g of uncaria hook, 4.0 g of the atractylodes lancea rhizome, 4.0 g of poria sclerotium, 3.0 g of Japanese angelica root, 3.0 g of cnidium rhizome and 1.5 g of glycyrrhiza (Fig. 2). The usual adult dose of yokukansan is 7.5 g taken orally in 2 to 3 divided doses daily before or during meals. Dosage may be adjusted based on age, body weight, and symptoms. Its indications include nervous disorders such as neurosis and insomnia, as well as pediatric use for crying at night and bad temper. In recent years, it has become frequently used and has been shown to be effective for behavioral and psychological symptoms of dementia.^[20–23]^

In the present case, prophylactic treatment of migraine without aura using yokukansan was effective primarily for menstrual migraines and its effects were persistent. It also reduced migraines associated with changes in weather and atmospheric pressure, although it did not eliminate them altogether. Several similar articles have been published in Japanese journals on the efficacy of yokukansan for migraine prophylaxis, but the only such articles published in international journals are our previous case report and one more case^[18,19]^ and a single clinical trial protocol investigating yokukansan for headache due to overuse of medications.^[24]^ We decided to publish this case report because we believe a second case report of markedly effective yokukansan therapy in an international journal would be valuable.

Migraines have a substantial negative impact on daily life and daily activities and are common among women in their 20s to 40s. Common triggers include the menstrual cycle, stress relief, excessive or insufficient sleep, changes in weather or atmospheric pressure, and hunger, but the mechanism of onset is not yet fully understood. Serotonin in platelets released into blood vessels in response to certain stimuli was previously believed to cause brain blood vessels to constrict, and subsequent dilation of these vessels caused headache. However, more recently proposed theories include central sensitization theory, which proposes that the cause of headaches is related to the brainstem or diencephalon (areas where migraines start), and trigeminovascular theory, which proposes that the cause of headaches lies in the trigeminal nerve endings and cerebral vasculature because release of vasoactive neuropeptides such as substance P, neurokinin A and calcitonin gene-related peptides in response to certain stimuli acting on trigeminal nerve endings distributed throughout the cerebral vasculature cause dilation of blood vessels in the brain along with sterile neurogenic inflammation, and this inflammatory response causes repeated vasodilation. Genetic factors have also been proposed. Furthermore, studies have shown that the glutamate concentration in plasma or cerebrospinal fluid increases during a migraine attack, and the glutamate concentration in platelets is also high, particularly in migraine with aura. Glutamate and the activation of glutamate receptors have also been implicated in cortical spreading depression, which is related to migraine attacks and auras.^[25–33]^

Migraine therapy consists of 2 fundamental elements: abortive therapy for acute attacks and prophylactic therapy used while the patient is not experiencing attacks. Drugs frequently used for prophylactic therapy are beta-blockers, calcium channel blockers, angiotensin-converting enzyme inhibitors/angiotensin II receptor antagonists, anticonvulsants, antidepressants, and *kampo* drugs. The mechanisms by which these drugs work are unclear, but it is believed that beta-blockers attenuate the dilation of cerebral blood vessels and suppress the release of serotonin; calcium channel blockers attenuate the constriction of cerebral blood vessels, reduce cortical spreading depression, increase vascular permeability and block 5-HT2 receptors; anticonvulsants activate glutamate decarboxylase and inhibit GABA (γ-aminobutyric acid) aminotransferase, stabilize cell membranes of neurons, and stimulate inhibitory cerebral activity through serotonin metabolism; and antidepressants suppress serotonin reuptake, block 5-HT2 receptors, and stimulate the descending pain suppression pathway.

Due to the limited clinical use of *kampo* drugs, and specifically yokukansan, for migraine prophylaxis to date, the mechanism of action remains unclear. However, animal studies of yokukansan that do not specifically investigate its use for headaches have found that it inhibits glutamate release,^[34]^ activates glutamate transporters and corrects glutamate reuptake,^[35]^ suppresses excessive release into extracellular fluid,^[36]^ has a partial agonistic effect on 5-HT1A receptors,^[37,38]^ downregulates 5-HT2A receptors,^[39]^ mediates GABA_A_-benzodiazepine receptor complex,^[40]^ suppresses secretion of orexin-A,^[41]^ and has anti-inflammatory effects.^[42,43]^ One crude drug in yokukansan, uncaria hook, is an indole alkaloid that contains gessoschizine methyl ether, a substance that acts on various serotonin receptors. It has been shown to have partial agonistic activity against 5-HT1A receptors and antagonistic activity against the 5-HT2A, 5-HT2B, 5-HT2C, and 5-HT7 receptors.^[38,44]^ We believe that yokukansan itself, or the crude uncaria hook of the yokukansan drug, prevented migraines in this case through these multifaceted mechanisms.

However, more research is warranted to establish the benefit of yokukansan for migraine prophylaxis. Specifically, the appropriate dose, duration of treatment, method of administration (continuous or intermittent), and patient characteristics associated with successful migraine therapy with yokukansan must be determined. Yokukansan shows promising effectiveness for migraine prophylaxis and could become a useful treatment in countries around the world. Large double-blind, placebo-controlled clinical trials of yokukansan and other *kampo* drugs should be conducted.

## 4. Conclusions

We found that prophylactic therapy with the *kampo* drug yokukansan, which is used for migraine without aura, was particularly effective for menstrual migraines. Yokukansan could be useful for migraine prophylaxis in countries all over the world and should be investigated in a large clinical trial as soon as possible.

## Author contributions

**Conceptualization:** Hisanao Akiyama.

**Data curation:** Hisanao Akiyama.

**Investigation:** Hisanao Akiyama.

**Project administration:** Hisanao Akiyama.

**Writing – original draft:** Hisanao Akiyama.

**Writing – review & editing:** Hisanao Akiyama, Yasuhiro Hasegawa, Yoshihisa Yamano.
